# Hematology and biochemistry of critically endangered radiated tortoises (*Astrochelys radiata*): Reference intervals in previously confiscated subadults and variability based on common techniques

**DOI:** 10.1371/journal.pone.0264111

**Published:** 2022-03-14

**Authors:** Maris Brenn-White, Bonnie L. Raphael, Ny Aina Tiana Rakotoarisoa, Sharon L. Deem

**Affiliations:** 1 Saint Louis Zoo Institute for Conservation Medicine, Saint Louis, Missouri, United States of America; 2 Turtle Survival Alliance, Charleston, South Carolina, United States of America; 3 Wildlife Conservation Society, Bronx, New York, United States of America; 4 Turtle Survival Alliance, Antananarivo, Madagascar; Universitat Autònoma de Barcelona, SPAIN

## Abstract

Madagascar’s radiated tortoises (*Astrochelys radiata*) are critically endangered, threatened by illegal collection, and confiscated in alarming numbers in recent years. Robust population- and technique-specific hematology and biochemistry reference intervals are valuable yet heretofore missing tools for triage, rehabilitation, and reintroduction of confiscated radiated tortoises. We determined reference intervals in 120 previously confiscated, clinically healthy subadult radiated tortoises living under human care within their native habitat at the Tortoise Conservation Center (TCC). Specific analytes measured were manual packed cell volume, total solids, white blood cell (WBC) count and differentials, and biochemistry analytes using a point of care system. To evaluate the effects of different commonly used techniques on these analytes, we compared results between two venipuncture sites (subcarapacial sinus and brachial vein) and three different WBC quantification methods (Natt and Herrick, Leukopet, and slide estimate). Reference intervals were narrower for most analytes, and sodium and potassium were qualitatively higher in the TCC population compared to previously published values from radiated tortoises housed in North American institutions. Creatine kinase, aspartate aminotransferase, glucose and inorganic phosphorus were all significantly greater in brachial samples than in subcarapacial samples. There was poor agreement and evidence of constant and/or proportional bias between all WBC quantification methods. Differences based on time of sample collection were incidentally found in some analytes. These results highlight the need for considering technique, demographic, and environmental factors in creating and applying reference intervals, and contribute foundational knowledge for improving care of radiated tortoises throughout the confiscation-to-release pathway.

## Introduction

Radiated tortoises (*Astrochelys radiata*) are critically endangered chelonians endemic to the spiny forests of southern Madagascar [1]. Habitat loss and poaching for trade and food are considered the primary threats to conservation of the species [1–5]. The scale of poaching is reflected in the massive confiscations of radiated tortoises that have taken place in recent years with over 10,000 tortoises recovered in a single 2018 event [6,7]. Over 24,000 confiscated radiated tortoises are currently in rehabilitation facilities in Madagascar, all managed by the Turtle Survival Alliance (TSA), the primary organization responsible for the care of rescued tortoises. Along with measures to minimize illegal collection, successful treatment, rehabilitation, and reintroduction of these and future confiscated radiated tortoises are critical to reversing the steep decline of this species in the wild.

Baseline biomedical information is essential to ensuring effective rehabilitation and safe reintroduction of wildlife, including chelonian species [8–10]. Hematology and biochemistry reference intervals based on clinically healthy tortoises are necessary for improving treatment of confiscated animals, selecting healthy candidates for release, and making evidence-based decisions about triage in confiscation events if and when resources allow [11–15]. Species and population-specific reference intervals also provide foundational information for subsequent research evaluating the efficacy of rehabilitation protocols and how these analytes may vary under different environmental conditions.

Robust hematology and biochemistry reference intervals for radiated tortoises have not previously been determined. Blood samples from more than 40 healthy individuals from a comparable reference population are recommended to establish de novo reference intervals with an absolute minimum of 20 individuals and 120 or more considered ideal [16–18]. To date, hematology and biochemistry research published for this species has been descriptive and based on sample sizes below 19 [19,20]. Additionally, prior published values and unpublished reference intervals have been based entirely on mixed age groups of individuals bred and/or managed within North American institutions [19–21]. As hematology and biochemistry values of reptile species change across demographic and environmental factors such as ambient temperature, humidity, rainfall patterns, food availability, and diet, there may be important differences between previously available hematology and biochemistry data and values in previously-confiscated tortoises being rehabilitated and conditioned for release in their native habitat [22–24].

Hematology and biochemistry values can vary not only by population, but also by sample collection and analytical methods. Choice of venipuncture site alone or in combination with lymph contamination has been shown to alter hematologic and biochemical analytes in a number of chelonian species [25–31]. Similarly, great variability in leukocyte counts using different quantification methods has been demonstrated in Galápagos tortoises (*Chelonoidis spp*.), eastern box turtles (*Terrapene carolina carolina*), and marine turtle species [32–36]. Veterinary and para-veterinary practitioners working with radiated tortoises may need to choose from multiple venipuncture sites and analytic methods based on patient size and temperament, practitioner training, cost and availability of supplies, and field conditions. Understanding how hematology and biochemistry analytes vary by technique is required to accurately apply population-specific reference intervals to interpretation of individual results.

To address these knowledge gaps in radiated tortoise health, our study had three objectives: 1) establish population-specific hematology and biochemistry reference intervals for previously confiscated, clinically healthy subadult radiated tortoises living under human care within their natural range; 2) evaluate differences in hematology and biochemistry values in blood from subcarapacial and brachial venipuncture sites within this population (between-animal comparison); and 3) evaluate agreement between three common leukocyte quantification methods within this population (within-animal comparison).

## Materials and methods

## Study site & population

In February of 2020, we examined and sampled radiated tortoises housed at the TCC, a TSA rehabilitation and captive care facility located in the Ala Mahavelo spiny forest in southern Madagascar within the radiated tortoise’s current range [5]. For security reasons, GPS coordinates of the TCC cannot be provided.

We selected study subjects from a population of 1,000 juvenile and subadult radiated tortoises (carapacial length 15–26 cm) [37] undergoing pre-release health screening. All tortoises were illegally collected from their native habitat, confiscated by law enforcement, and placed under TSA management for rehabilitation and conditioning for release. Time at the TCC prior to sampling was between six months and two years for all individuals. Only subadults were included in this study as this is the age group targeted for this and subsequent releases. Sex of these tortoises is unknown as sexual dimorphism is not reliably apparent in this age group. The tortoises are housed year-round in 1000m^2^ open pens filled with native vegetation and natural substrate. Diets are supplemented with locally cultivated plants and native vegetation foraged by neighboring communities.

### Study subjects, design, & sample collection

To allow time for same-day laboratory analyses, we screened the first 8 to 18 tortoises examined between 6:30 AM and 11:00 AM each day for inclusion in this study. Occasional afternoon screening and sampling of tortoises occurred between 4:00 PM and 5:30 PM when time permitted. Screening in the field consisted of a physical examination by a veterinarian. This included routine visual inspection and palpation, oral and cloacal examination, and coelomic palpation as size and temperament allowed. We collected oral-cloacal swabs for post-hoc screening for *Mycoplasma* spp., herpesviruses, and the intranuclear coccidian parasite of Testudines via polymerase chain reaction conducted by the Wildlife Conservation Society Molecular Lab as previously described [10,38–41]. Only tortoises that were apparently healthy on physical examination and negative for all pathogens via polymerase chain reaction were included in the study. We sampled 136 tortoises, with 120 samples of sufficient volume and gross quality for analysis. All handling and sample collection was performed according to Saint Louis Zoo’s Institutional Animal Care and Use Committee application 19–09 and Direction de la Gestion des ressources naturelles renouvelables et des ecosystemes research permit 026/20/MEDD/SG/DGEF/DGRNE.

We manually restrained tortoises [42] for blood collection from either the subcarapacial sinus (SC) or brachial vein using pre-heparinized 1 ml or 3 ml syringes with 0.64 mm (23 g) or 0.51 mm (25 g) needles of 25.4 mm (1”) or 38.1 mm (1.5”) in length as appropriate for patient size and temperament [43–45]. We collected no more than 3 ml of blood per tortoise, which was equivalent to less than 0.5% of body weight [45], and discarded samples with evidence of lymph contamination or clotting on gross examination. Using fresh blood directly from the syringe, we made two blood smears on glass slides and fixed them for more than one minute in methanol after air-drying. We removed the needle from the syringe and immediately transferred the remaining blood to lithium heparin blood collection tubes (Becton, Dickinson and Company, Franklin Lakes, New Jersey 07417, USA), which were filled > 2/3 of capacity and kept in coolers until analysis.

### Hematology and biochemistry analyses

We performed all sample processing and analyses at an on-site field laboratory within 0.5 to 11 hours of sample collection with the exception of review of blood smears, which were stained the same day with a modified Wright-Giemsa stain (Dip Quick Stain, Jorgensen Laboratories, Loveland, Colorado 80538, USA) and evaluated within 14 days. We measured packed cell volume (PCV) using non-heparinized microhematocrit tubes centrifuged for six minutes at 1534 G (3,500 rpm) using hematocrit-specific adaptors (Crit Carrier, LW Scientific, Lawrenceville, GA 30045, USA) in a portable angled centrifuge (E8, LW Scientific, Lawrenceville, GA 30045, USA). We measured total solids (TS) in plasma from the microhematocrit tube using a manually calibrated, handheld clinical refractometer (J-351, Jorgensen Laboratories, Loveland, Colorado 80538, USA). We performed biochemistry analyses on whole blood using a VetScan® VS2 chemistry analyzer (Abaxis Inc., Union City, California 94584, USA) with VetScan Avian Reptilian Profile Plus rotors (Abaxis Inc., Union City, California 94584, USA). The biochemical panel included: albumin (Alb), bile acids (BA), aspartate aminotransferase (AST), total calcium (Ca), creatinine kinase (CK), glucose (GLU), potassium (K), sodium (Na), inorganic phosphorus (P), globulin (Glob), total protein (TP), and uric acid (UA).

We performed total white blood cell (WBC) quantification or estimates on each sample using three techniques. The Natt and Herrick’s method (NH) was performed using Natt-Herricks-TIC® 1:200 staining kits (Bioanalytic GmbH, D79224, Umkirch, Germany) per manufacturer instructions, charged into a Neubauer hemocytometer and calculated as: WBC x 10^9^/L = number of WBC counted in 9 squares x 0.2/ 9. The Leukopet method (LP) was performed using Avian Leukopet^TM^ Kits (Vetlab Supply, Palmetto Bay, FL) per manufacturer instruction, charged into a Neubauer hemocytometer and calculated as: WBC x 10^9^/L = (number of eosinophilic staining cells [heterophils and eosinophils] counted in 18 squares x 1.1 x 16 x 0.1)/(% heterophils + % eosinophils determined from differential counts). For NH and LP, samples were incubated at room temperature with stain for five and ten minutes, respectively, and all were allowed to settle in the hemocytometer in a humidity chamber for three minutes prior to reading. Total WBC estimates (EST) were performed on the highest quality blood film from each tortoise by counting all leukocytes in ten fields of the monolayer using a 40x objective lens and calculated as: WBC x 10^9^/L = (average number of leukocytes per field) x (objective power)^2^ x 0.001 [46]. We performed 100-cell leukocyte differentials on the same blood film using a 100x objective lens under oil immersion. We calculated method-specific absolute values for each WBC type by multiplying the percentage of a given cell type from the differential by the total leukocyte count from each WBC quantification method. All NH, EST, and differentials were performed by one observer. All LP were performed by a second observer after verifying that LP results performed by both observers were identical on a subset of samples.

### Statistical analyses

All statistical analyses were performed in R v.4.0.3 [47] using the RStudio v. 1.3.1093 interface [48] unless specified otherwise. For all statistical tests, p < 0.05 was considered significant with the exception of the Anderson-Darling test for Gaussian distribution for reference interval calculation, where p < 0.3 was used per recommendations for samples of this size [49].

#### Pre-analysis data preparation

Twenty samples were excluded from all analyses for gross lymph contamination (SC: n = 14/94 [14.8%]; brachial: n = 6/54 [11.1%]) and eight for clotting (SC: n = 0/94 [0.0%]; brachial: n = 8/54 [14.8%]). Slide quality issues (n = 9) and cell clumping on LP (n = 8) precluded additional samples from applicable WBC analyses. For values that fell outside of the dynamic range of the VetScan VS2 analyzer, we substituted the reported qualitative values with the quantitative value closest to the analyzer threshold to reduce the bias that would be introduced by removing these high and low values from the dataset [50]. Substitutions were made as follows: Ca– 4.01 for > 4.00 mmol/L; K– 8.6 for > 8.5 mmol/L; TP– 19 for < 20 g/L; and UA– 17 for < 18 μmol/L.

We evaluated the distribution of each hematologic and biochemical analyte by inspection of histograms, the Anderson-Darling test for Gaussian distribution [51], and a distribution-free test for symmetry [52]. For data that had a non-Gaussian distribution, we applied a Box-Cox transformation [53] and repeated evaluation of the distribution as above. We used Tukey’s interquartile range to identify statistical outliers on untransformed Gaussian data or on Box-Cox transformed data for non-Gaussian data. We removed only those outliers that could be attributed to sample, handling, or analytical error. After removing an outlier, we repeated the above procedures until no further outliers warranting removal were identified. Outliers removed from applicable analyses were: NH– 18.87 WBC x 10^9^/L, LP– 28.86 WBC x 10^9^/L, EST– 29.60 WBC x 10^9^/L, and proportion of eosinophils– 0.15. Values altered specifically by hemolysis or extended time from sample collection to analysis were identified and handled as described below.

#### Effects of venipuncture site, sample artifacts, and time of sample collection (between-animal)

To identify potential confounds and presence of sample artifacts, we tested the associations between venipuncture site (SC, brachial), hemolysis (0, 1+, 2+, 3+), time of sample collection (AM, PM), and time from sample collection to biochemistry analysis using Kruskal-Wallis, Wilcoxon rank sum, and chi-square tests. As significant relationships existed between hemolysis and both venipuncture site and time to analysis as well as between time of sample collection and time to analysis, we used univariate generalized linear models (GLM) fit by the glm function [47] to simultaneously evaluate the effects of these four variables on our variables of interest. We fit separate models with each hematological analyte as the response and venipuncture site, hemolysis, and time of sample collection as predictors. We evaluated effects on biochemical analytes in the same manner with time to analysis included as an additional predictor. Model assumptions were checked using residual vs. fitted, normal Q-Q, scale-location, and residual vs. leverage plots. When model fit was poor, Box-Cox transformation was used. If this failed to provide sufficient fit, we performed stratified non-parametric tests with Hommel corrected p-values for each predictor-response pair. We conditionally averaged the best fit models defined as ΔAICc < 2. We determined the threshold at which hemolysis and/or time to analysis no longer exhibited a statistically significant effect on a given hematological or biochemical analyte by iteratively removing samples with the greatest hemolysis and/or time to analysis from the dataset and rerunning the above models or tests. We additionally evaluated differences in sample quality (hemolysis, gross clotting, and gross lymph contamination) based on venipuncture site using chi-square and Fisher exact tests.

#### Reference interval & descriptive statistics

For a given analyte, we excluded samples determined to be affected by hemolysis or time to analysis as described above from reference interval calculation. For all analytes, samples collected in both the AM and PM were included in reference interval calculation as we considered variation based on time of sample collection to represent physiologic variation rather than pathology or handling artifact. Additionally, the number of samples collected in the PM (n = 13) was below the threshold recommended by American Society of Veterinary Clinical Pathology (ASVCP) guidelines for partitioning of reference intervals [16]. We used Reference Value Advisor v. 2.1 [54] to calculate reference intervals, 90% confidence intervals (CI) of the lower and upper reference interval limits, and the descriptive statistics mean, standard deviation, and median for each hematological and biochemical analyte. We calculated these values separately for each WBC quantification method and for each venipuncture site when venipuncture site was found to have an effect on an analyte. We calculated reference intervals and CIs according to the ASVCP guidelines [16] as follows: non-parametric methods for reference intervals and CIs when n = 120; robust method for reference intervals and bootstrap method for CIs when 40 ≤ n < 120 and distribution was symmetric; non-parametric method for reference intervals and bootstrap method for CIs when 40 ≤ n < 120 and distribution was not symmetric; and robust method for reference intervals and bootstrap method for CIs when 20 ≤ n < 40 and distribution was symmetric. When n < 120, we used Box-Cox transformed data for reference intervals and CI calculations of non-Gaussian distributed analytes when transformation improved distribution symmetry. Reference intervals were not calculated when n < 20 or when 20 ≤ n < 40 and analyte distribution was neither Gaussian nor symmetric.

#### Agreement between WBC quantification method (within-animal)

We evaluated agreement in total WBC count from the NH, LP, and EST methods using Passing-Bablok (PB) regression and Bland Altman plots. Passing-Bablok assumes a high positive correlation between the diagnostic methods being evaluated [55,56], which we tested using Kendall’s tau. We considered the methods in statistical agreement per PB when the 95% CI for the slope, the indicator of constant bias, contained one and the 95% CI for the y-intercept, the indicator of proportional bias, contained zero. We used Bland-Altman plots, estimates of bias (defined as the mean difference between methods), and limits of agreement (defined as the bias ± 1.96 times the standard deviation of the differences) to further evaluate agreement, systematic bias, and proportional bias [57]. As it has been proposed that LP results vary according to lymphocyte and heterophil proportions [24], we used Kendall’s tau to test for correlation between proportions of these cell types and the difference in total WBC count for each method pair.

## Results

### Reference intervals & descriptive statistics

Reference intervals calculated for hematology and biochemistry analytes of tortoises are reported in Tables 1 and 2 respectively. See S1 Table for biochemistry reference intervals and descriptive statistics presented in United States conventional units.

**Table 1:** Subadult radiated tortoise hematology reference intervals and descriptive statistics based on clinically healthy individuals living in a rehabilitation setting within their natural range.

*Analyte*	*n*	*Mean*	*SD*	*Median*	*Min*	*Max*	*p*	*Dist*.	*RI Method*	*RI*	*LRL 90% CI*	*URL 90% CI*	*Data excluded*
PCV (L/L)	120	0.23	0.03	0.22	0.18	0.32	0.001	NG	NP	0.18–0.30	0.18–0.19	0.27–0.32	N/A
***Natt Herrick***													
Total WBC (10^9^/L)	119	7.50	2.43	7.44	2.11	13.32	0.470	G	R	2.59–12.27	1.98–3.16	11.58–12.91	1 outlier
Heterophil (10^9^/L)	110	1.63	1.00	1.41	0.11	5.82	<0.001	NG	RT	0.33–4.20	0.26–0.43	3.56–4.94	2 outliers
Lymphocyte (10^9^/L)	110	4.66	1.82	4.56	1.15	11.19	0.092	NG	RT	1.66–8.72	1.43–1.96	8.03–9.39	2 outliers
Monocyte (10^9^/L)	110	0.46	0.31	0.41	0.04	1.70	<0.001	NG	NP	0.06–1.21	0.04–0.07	1.02–1.70	2 outliers
Eosinophil (10^9^/L)	110	0.22	0.21	0.17	0.00	1.18	<0.001	NG	NP	0.00–0.81	0.00–0.00	0.63–1.18	2 outliers
Basophil (10^9^/L)	110	0.58	0.43	0.48	0.00	1.91	<0.001	NG	NP	0.03–1.73	0.00–0.06	1.41–1.91	2 outliers
***Leukopet***													
Total WBC (10^9^/L)	110	8.76	5.03	7.85	0.87	26.19	<0.001	NG	NP	2.00–22.89	0.87–3.16	18.92–26.19	1 outlier
Heterophil (10^9^/L)	102	1.63	0.91	1.41	0.50	5.76	<0.001	NG	NP	0.66–4.84	0.50–0.73	3.35–5.76	2 outliers
Lymphocyte (10^9^/L)	102	5.59	3.88	4.23	0.82	18.59	<0.001	NG	NP	1.19–16.53	0.82–1.50	14.33–18.59	2 outliers
Monocyte (10^9^/L)	102	0.52	0.45	0.36	0.04	1.97	<0.001	NG	RT	0.07–1.97	0.05–0.08	1.56–2.48	2 outliers
Eosinophil (10^9^/L)	102	0.21	0.19	0.17	0.00	0.90	<0.001	NG	NP	0.00–0.83	0.00–0.00	0.60–0.90	2 outliers
Basophil (10^9^/L)	102	0.63	0.58	0.49	0.00	3.45	<0.001	NG	NP	0.02–2.54	0.00–0.05	1.69–3.45	2 outliers
***Slide Estimate***													
Total WBC (10^9^/L)	107	11.15	3.41	10.72	4.80	21.12	0.001	NG	NP	5.60–20.62	4.80–6.40	17.88–21.12	1 outlier
Heterophil (10^9^/L)	106	2.43	1.59	2.09	0.32	9.72	<0.000	NG	NP	0.61–7.33	0.32–0.84	5.57–9.72	2 outliers
Lymphocyte (10^9^/L)	106	6.88	2.38	6.63	1.23	17.61	0.042	NG	NP	3.09–12.16	1.23–3.92	10.31–17.61	2 outliers
Monocyte (10^9^/L)	106	0.68	0.47	0.61	0.06	2.40	<0.001	NG	R	0.08–1.95	0.06–0.12	1.69–2.22	2 outliers
Eosinophil (10^9^/L)	106	0.30	0.26	0.24	0.00	1.05	<0.001	NG	NP	0.00–0.99	0.00–0.00	0.92–1.05	2 outliers
Basophil (10^9^/L)	106	0.86	0.69	0.65	0.00	4.51	<0.001	NG	NP	0.04–2.83	0.00–0.11	2.08–4.51	2 outliers
***Differential***													
Heterophil	111	0.21	0.10	0.19	0.05	0.46	<0.001	NG	NP	0.07–0.44	0.05–0.09	0.39–0.46	1 outlier
Lymphocyte	111	0.62	0.13	0.64	0.22	0.88	0.063	NG	NP	0.36–0.84	0.22–0.41	0.80–0.88	1 outlier
Monocyte	111	0.06	0.04	0.06	0.01	0.17	<0.001	NG	NP	0.01–0.20	0.01–0.01	0.13–0.17	1 outlier
Eosinophil	111	0.03	0.02	0.03	0.00	0.12	<0.001	NG	NP	0.00–0.10	0.00–0.00	0.07–0.12	1 outlier
Basophil	111	0.08	0.05	0.07	0.00	0.22	0.001	NG	NP	0.01–0.20	0.00–0.01	0.18–0.22	1 outlier
Het:Lymph Ratio	111	0.39	0.26	0.31	0.06	1.56	<0.001	NG	NP	0.08–1.09	0.06–0.13	0.90–1.56	1 outlier

CI = confidence interval, Dist. = distribution, G = Gaussian, hem = hemolysis (0, 1+, 2+, 3+), Het:Lymph = heterophil:lymphocyte, LRL = lower reference limit, NG = Non-Gaussian, NP = Non-parametric, p = Anderson-Darling p value, R = Robust, RT = Robust on Box Cox transformed data, SD = standard deviation, URL = upper reference limit, WBC = white blood cell. α = 0.3 for distribution tests per ASVCP Guidelines 2020. Samples sizes vary between and within methods as samples were excluded from affected analyses based on slide and Leukopet quality issues in addition to the presence of outliers.

**Table 2:** Subadult radiated tortoise biochemistry reference intervals and descriptive statistics based on clinically healthy individuals living in a rehabilitation setting within their natural range.

*Analyte*	*VS*	*n*	*Mean*	*SD*	*Median*	*Min*	*Max*	*p*	*Dist*.	*RI Method*	*RI*	*LRL 90% CI*	*URL 90% CI*	*Data excluded*
TS (g/L)	All	120	38	7	39	23	58	0.012	NG	NP	25–51	23–27	48–58	N/A
TP (g/L)	All	120	29	5	29	19	43	0.210	NG	NP	19–42	19–21	37–43	N/A
AST (U/L)	All	120	81	43	68	31	342	<0.001	NG	NP	39–218	31–44	166–342	N/A
AST (U/L)	SC	80	74	34	65	31	245	<0.001	NG	NP	38–209	31–41	120–245	N/A
AST (U/L)	Br	40	95	55	81	47	342	<0.001	NG	NP	47–339	47–51	194–342	N/A
CK (U/L)	All	53	660	626	487	193	4011	<0.001	NG	RT	201–2455	184–229	1726–3618	time to analysis ≥ 5 hrs; hem > 0
CK (U/L)	SC	39	478	235	452	193	1358	0.008	NG	RT	190–1072	0–211	898–1281	time to analysis ≥ 5 hrs; hem > 0
CK (U/L)	Br	14	1165	1016	912	310	4011	0.008	NG	N/A	N/A	N/A	N/A	time to analysis ≥ 5 hrs; hem > 0
Uric acid (μmol/L)	All	120	23	8	17	17	48	<0.001	NG	NP	17–42	17–17	42–48	N/A
Glucose (mmol/L)	All	90	3.2	0.7	3.2	1.9	5.8	0.477	G	R	1.8–4.5	1.6–2.1	4.2–4.7	time to analysis ≥ 5 hrs
Glucose (mmol/L)	SC	57	2.9	0.6	2.9	1.9	4.1	0.417	G	R	1.8–4.0	1.7–2.1	3.8–4.2	time to analysis ≥ 5 hrs
Glucose (mmol/L)	Br	33	3.6	0.7	3.6	2.0	5.8	0.183	NG	RT	2.3–5.2	2.1–2.7	4.6–5.7	time to analysis ≥ 5 hrs
Total Ca (mmol/L)	All	120	2.97	0.32	2.94	2.15	4.02	0.049	NG	NP	2.35–3.72	2.15–2.40	3.44–4.02	N/A
Phos (mmol/L)	All	120	1.03	0.23	1.00	0.71	1.81	<0.001	NG	NP	0.74–1.61	0.71–0.77	1.49–1.81	N/A
Phos (mmol/L)	SC	80	1.00	0.16	0.97	0.71	1.52	0.001	NG	NP	0.74–1.49	0.71–0.77	1.32–1.52	N/A
Phos (mmol/L)	Br	40	1.10	0.26	1.07	0.74	1.81	<0.001	NG	NP	0.74–1.81	0.74–0.77	1.61–1.81	N/A
Ca:Phos Ratio	All	120	3.9	0.8	3.8	2.1	5.7	0.639	G	R	2.3–5.4	2.1–2.5	5.2–5.6	N/A
Ca:Phos Ratio	SC	80	4.0	0.8	3.9	2.2	5.7	0.609	G	R	2.5–5.5	2.3–2.7	5.3–5.7	N/A
Ca:Phos Ratio	Br	40	3.6	0.8	3.5	2.1	5.2	0.858	G	R	2.0–5.2	1.7–2.4	4.9–5.5	N/A
K (mmol/L)	All	77	6.4	0.9	6.5	4.3	8.4	0.553	G	R	4.5–8.2	4.2–4.8	7.9–8.5	time to analysis ≥ 8 hrs
Na (mmol/L)	All	120	145	11	144	124	169	0.001	NG	NP	128–168	124–130	166–169	N/A

Results stratified by venipuncture site where statistically significant differences were found via generalized linear models. AST = aspartate transaminase, Br = brachial, Ca = calcium, CI = confidence interval, CK = creatine kinase, Dist. = distribution, G = Gaussian, hem = hemolysis (0, 1+, 2+, 3+), K = potassium, LRL = lower reference limit, Na = sodium, NG = Non-Gaussian, NP = Non-parametric, p = Anderson-Darling p-value, Phos = phosphorus, R = Robust, RT = Robust on Box Cox transformed data, SC = subcarapacial, SD = standard deviation, time to analysis = time from sample collection to biochemistry analysis, TP = total protein, TS = total solids, UA = uric acid, URL = upper reference limit, VS = venipuncture site. α = 0.3 for distribution tests per ASVCP Guidelines 2020.

Reference intervals could not be calculated for three biochemistry analytes due to large proportions of the results falling outside of the dynamic range of the VetScan VS2 analyzer. All bile acid results (n = 120) were reported as < 35 μmol/L. Of 120 albumin results, 107 were reported as < 10 g/L with the remaining 13 results yielding a mean and standard deviation of 12 +/- 2 g/L, median of 11 g/L, and range of 10–17 g/L. As globulin concentration is calculated from total protein and albumin, only 13 values were reported for globulin with a mean and standard deviation of 24 +/- 3 g/L, median of 23 g/L, and range of 17–28 g/L.

### Effects of venipuncture site (between-animal)

No significant effects on hematology analytes were found. All biochemistry analytes on which venipuncture site had a significant effect were greater in brachial samples than in subcarapacial samples. These included: AST (GLM: β = 19.9 [3.35–36.5] U/L, p = 0.018), CK (GLM: β = 893 [564–1222] U/L, p < 0.001), Glu (GLM: β = 0.630 [0.851–0.410] mmol/L, p < 0.001), and Phos (GLM: β = 0.107 [0.191–0.0222] mmol/L, p = 0.013). Conditionally averaged best-fit GLMs evaluating the effects of venipuncture site along with time of sample collection, hemolysis, and time from sample collection to biochemistry analysis are reported in Table 3. Effects of venipuncture site on uric acid, proportion of eosinophils, and presence of immature lymphocytes were evaluated with non-parametric tests due to inability to obtain appropriate GLM fit and no significant effects were identified. Gross clotting and hemolysis levels greater than 1+ were more frequent in brachial samples than SC samples (chi-square p < 0.001 and Fisher exact p < 0.001, respectively). There was no significant difference in gross lymph contamination (chi-square p = 0.517) between venipuncture sites.

**Table 3:** Effects of venipuncture site, hemolysis, and time from sample collection to biochemistry analysis on hematology and biochemistry analytes in clinically healthy subadult radiated tortoises living in a rehabilitation setting within their natural range.

RESPONSE	PREDICTORS									
	Venipuncture site: Br (ref = SC)		Hemolysis: 1+ (ref = 0)		Hemolysis: 2+ (ref = 0)		Venipuncture time: PM (ref = AM)		Time to chemistry analysis (hrs)	
*Analyte*	β *(95% CI)*	*p*	β *(95% CI)*	*p*	β *(95% CI)*	*p*	β *(95% CI)*	*p-value*	β *(95% CI)*	*p*
AST (U/L)	19.9 (3.35–36.5)	0.018*	-	-	-	-	12.11 (-12.8–37.0)	0.700	0.401 (-2.59–6.03)	0.359
CK (U/L)	893 (564–1220)	<0.001*	356 (33.1–679)	0.031*	1021 (587–1456)	<0.001*	-166 (-652–319)	0.502	-93.3 (-176–-10.9)	0.026*
Glucose (mmol/L)	0.630 (0.410–0.851)	<0.001*	-	-	-	-	-	-	-0.102 (-0.159–-0.0448)	<0.001*
Total Ca (mmol/L)	-	-	-6.28e4 (-0.126–0.124)	0.992	-0.149 (-0.307–9.85e3)	0.066	-0.226 (-0.406–-0.0471)	0.013*	-	-
Phos (mmol/L)	0.107 (0.0222–0.191)	0.013*	0.0747 (-9.31e3–0.159)	0.081	0.0960 (-0.0155–0.208)	0.091	-0.0394 (-0.160–0.0813)	0.522	8.59e3 (-0.0124–0.0296)	0.422
K (mmol/L)	-	-	-0.128 (-0.556–0.304)	0.562	0.352 (-0.191–0.896)	0.204	1.06 (0.431–1.68)	<0.001*	0.110 (0.00293–0.217)	0.044*
Heterophils	-	-	-	-	-	-	0.0769 (0.0153–0.139)	0.016*	N/A	N/A
Lymphocytes	0.0301 (0.0200–0.0802)	0.239	-	-	-	-	-0.111 (-0.191–-0.0315)	0.006*	N/A	N/A
Het:Lymph Ratio†	-0.0948 (-0.359–0.170)	0.482	-	-	-	-	0.580 (0.161–1.000)	0.007*	N/A	N/A

Results presented are the average of the best fit univariate generalized linear models (ΔAICc < 2) for each response analyte. Only models containing statistically significant predictors are presented. Br = brachial, AST = aspartate transaminase, Ca = calcium, CI = confidence interval, CK = creatine kinase, K = potassium, Na = sodium, p = Wald Z p-value, Phos = phosphorus, ref = reference value of categorical predictors, SC = subcarapacial,— = analyte not included in best fit model(s)

* = p < 0.05, † = analyte log transformed.

### Effects of time of sample collection (between-animal)

Time of sample collection did not have a significant effect on total WBC counts by any method, but the proportion of individual WBC types was significantly affected with PM samples being associated with increased heterophils (GLM: β = 0.0769 [0.0153–0.139], p = 0.016), decreased lymphocytes (GLM: β = -0.111 (-0.191–-0.0315), p = 0.006), and increased heterophil:lymphocyte ratios (GLM: β = 0.580 [0.161–1.000], p = 0.007) compared to AM samples. Of the biochemistry analytes, only Ca (GLM: β = -0.226 (-0.406–-0.0471) mmol/L, p = 0.013) and K (GLM: β = 1.06 [0.431–1.68] mmol/L, p < 0.001) were significantly affected.

### Agreement between WBC quantification method (within-animal)

**Fig 1:**
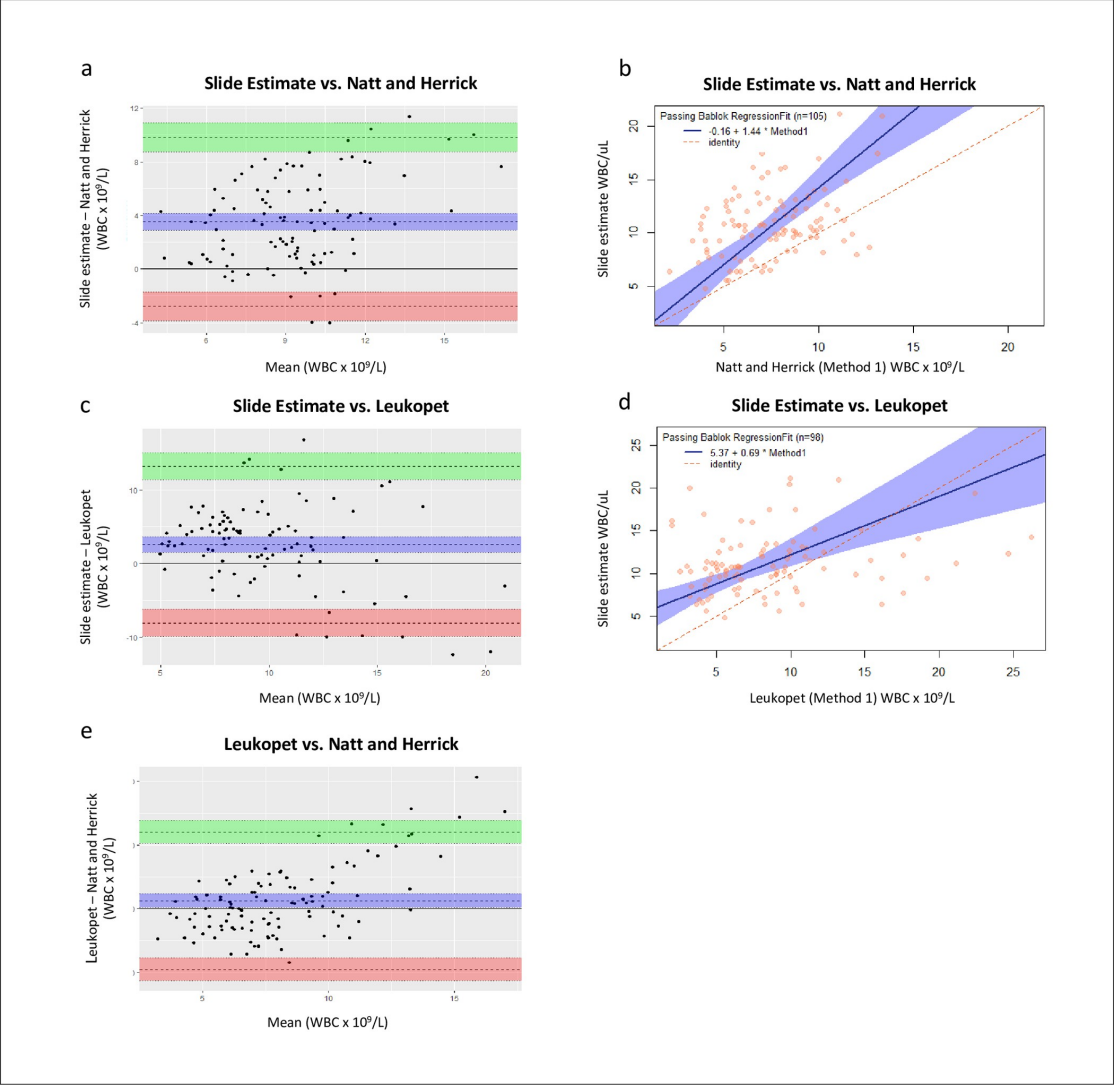
Agreement between WBC quantification methods in clinically healthy, subadult radiated tortoises. (a, c, e) Bland-Altman–solid line is the line of perfect agreement, dashed lines are mean difference between the methods (center), upper limit of agreement (upper), and lower limit of agreement (lower) with shading indicating associated 95% confidence intervals. (b, d) Passing-Bablok regression–dashed line is the line of perfect agreement, solid line is the regression line with shading indicating associated 95% confidence intervals. WBC = white blood cell.

There was poor statistical agreement between all method pairs based on PB regression and Bland Altman plots (Fig 1, Table 4) with limits of agreement wider than 12.00 WBC x 10^9^/L.

**Table 4:** Agreement between total white blood cell counts determined using the Natt and Herrick, Leukopet, and blood slide estimate methods in clinically healthy subadult radiated tortoises.

			Passing-Bablok		Bland-Altman		
*Methods compared*	*n*	*Kendall’s Tau (p-value)*	*Intercept (95% CI)*	*Slope (95% CI)*	*Bias (95% CI)*	*Lower LOA (95% CI)*	*Upper LOA (95% CI)*
* *	* *	* *	* *	* *	* *	* *	* *
Est vs. NH	105	0.218 (0.001)*	-0.16 (-3.02–3.12)	1.44 (1.03–1.83)	3.51 (2.89–4.13)	-2.81 (-3.88–-1.74)	9.83 (8.76–10.90)
Est vs. LP	98	0.167 (0.016)*	5.37 (3.10–7.52)	0.685 (0.415–1.02)	2.57 (1.49–3.66)	-8.04 (-9.90–-6.18)	13.19 (11.33–15.05)
LP vs. NH	109	0.017 (0.793)	N/A	N/A	1.26 (0.21–2.30)	-9.53 (-11.32–-7.74)	12.05 (10.25–13.84)

A slope with a CI that does not include one indicates proportional bias and an intercept with a CI that does not include zero indicates constant bias. Bias = the mean difference between the two methods, CI = confidence interval, Est = blood slide estimate, * = p < 0.05, LOA = limit of agreement defined as the bias + 1.96 times the standard deviation of the differences, LP = Leukopet, N/A = estimates invalid due to lack of positive correlation based on Kendall’s Tau, NH = Natt and Herrick, * = p < 0.05. Intercept, bias, and LOAs reported in WBC x 10^9^/L.

Bland Altman plots revealed systematic bias between each pair with the EST method yielding higher values on average than the LP method (bias = 2.57 [1.49–3.66] WBC x 10^9^/L), which in turn yielded higher values than the NH method (bias = 1.26 [0.21–2.30] WBC x 10^9^/L). Passing-Bablok revealed constant bias between LP and EST values (intercept = 5.37 [3.08–7.40] WBC x 10^9^/L) and proportional bias between EST and NH values (slope = 1.44 [1.03–1.83]), though the 95% CI approaches one. Results of the LP and NH methods failed the test for positive correlation prohibiting PB regression analysis. On Bland Altman plots, all pairs showed a trend toward larger differences as mean WBC counts increased. The difference between LP and NH values was positively correlated with lymphocyte proportion (τ = 0.332, p < 0.001) and negatively correlated with heterophil proportion (τ = -0.399, p < 0.001), as was the difference between LP and EST values (τ = 0.348, p < 0.001 and τ = -0.427, p < 0.001 respectively). The difference between NH and EST values was not significantly correlated with either lymphocyte proportion (τ = 0.0152, p = 0.814) or heterophil proportion (τ = -0.0313, p = 0.632).

## Discussion

Robust population and technique-specific hematology and biochemistry reference intervals are valuable yet previously missing tools throughout the confiscation-to-release pathway that is important to reversing radiated tortoise population declines. Low cost, low technology analytes such as PCV and TS have proven useful as triage criteria with avian species in large scale oil spills [58–60]. They could be similarly informative in directing resources during tortoise confiscation events, though measuring even these two analytes may be challenging during large scale confiscations in low resource settings. After triage and when resources allow, hematology and biochemistry reference intervals can be used to help guide clinical care of confiscated tortoises just as they are used for a wide variety of taxa in rehabilitation settings [13,61–64]. These reference intervals also contribute to evaluating the health status of tortoises prior to release, as was the context of this study, with the goal of identifying and removing tortoises with evidence of disease from the release population [10,65]. Lastly, baseline values in the pre-release population provide a foundation for future research aimed at evaluating and improving management at all stages of care from confiscation until release [10,14,66–68].

This study provides statistically robust population- and technique-specific reference intervals for clinically healthy radiated tortoises in the priority age group for reintroduction living in a managed setting within native habitat. While reference intervals reported here are largely comparable to previously reported ranges for radiated tortoises in North American institutions [19–21], some important differences are apparent. Potassium and Na are notably higher in this population, which is most likely attributable to differences in water balance and diet between these settings. Drought-adapted chelonians tolerate wide variation in circulating K, Na, and Cl levels [69–71], all of which have been found to be higher in free-living desert tortoises (*Gopherus agassizii*) during seasons with relatively low rainfall and where diets are high in K [23,71]. This study was performed at the end of an extended dry season and thus under conditions of decreased water availability and altered forage, which likely also contributed to the relative elevations in K and Na in this population. In general, reference intervals for this population are substantially narrower than reference intervals calculated by ZIMS, which combine values from multiple age groups, venipunctures sites, biochemistry analyzers, and seasons. When based on adequate sample sizes, narrower intervals provide increased sensitivity in detecting abnormalities and highlight the value of determining and applying reference intervals on a population- and technique-specific basis.

We additionally quantified the effects of venipuncture site on hematology and biochemistry analytes in radiated tortoises and, to the authors’ knowledge, provide the first direct comparison between SC and brachial venipuncture sites in any species. While jugular blood is generally considered the gold standard sample in chelonians [72], SC and brachial venipuncture are the two most accessible techniques in unsedated radiated tortoises across the age groups in this study. Statistically significant differences were observed in AST, CK, Glu, and Phos, all of which were greater in brachial samples than in SC samples. These differences were present independent of level of hemolysis, time from sample collection to biochemistry analysis, and time of venipuncture. Of these, the magnitude of differences in CK, Glu, and Phos exceeded the thresholds adopted by the ASCVP for creation of separate reference intervals for clinical use [73,74]. Venipuncture site-based differences in CK (GLM: β = 893 [564–1222] U/L) and Glu (GLM: β = 0.630 [0.851–0.410] mmol/L) are particularly relevant for evaluating the physiologic effects of field capture and handling techniques and accurately identifying hypoglycemia in emergency or triage scenarios.

Relatively increased AST and CK are most likely attributable to restraint technique and relatively increased, although minor, iatrogenic tissue trauma during brachial venipuncture. This is supported by greater proportions of brachial samples exhibiting gross clotting (n = 8/54 [14.8%], Fisher exact p < 0.001) and hemolysis levels greater than 1+ (n = 15/40 [37.5%], chi-square p < 0.001) as compared to SC samples (n = 0/94 [0.0%] and n = 6/80 [7.5%], respectively). The etiology of the relatively decreased Glu and Phos in SC samples is less clear. This finding is consistent with presumed or observed lymph dilution during SC venipuncture in other chelonian species [25,26,28]. While gross lymph contamination was noted in a slightly greater proportion of SC (n = 14/94 [14.8%]) compared to brachial (n = 6/54 [11.1%]) samples in this study, this difference was far from statistically significant (chi-square p = 0.517). Further, lymph contamination is a form of hemodilution that most commonly reduces PCV, WBC count, and TP [25,26,28–30], none of which were observed here. Alternatively, an undetected difference between tortoises in the SC and brachial sample groups may be responsible, though the overall study population is homogenous in terms of age, size, apparent health status, husbandry, and environmental conditions.

As with venipuncture site, choice of leukocyte quantification method has been shown to have marked impacts on results in all chelonian species in which comparison of methods has been performed [31–36]. Leukocyte results in this study demonstrated poor clinical agreement between all methods with LOAs ranging from 12.64–21.58 WBC x 10^9^/L and markedly different method-specific reference intervals. Width of the LOAs is due in part to random error inherent in manual leukocyte quantification methods even when performed by a single highly-trained observer. While imprecision has rarely been quantified, recent studies reported coefficients of variation of 8.2% using the LP method in eastern box turtles (*Terrapene carolina carolina*) [36] and 12% using the NH method in multiple avian species [75]. Despite the effects of random error, systematic and/or proportional biases are evident between all method pairs. On average, EST overestimated both LP (bias = 2.57 [1.49–3.66] x 10^9^/L) and NH (bias = 3.51 [2.89–4.13] WBC x 10^9^/L). This is consistent with comparisons between EST and NH in Galapagos tortoises (*Chelonoidis spp*.) [35], but contrary to studies comparing EST and LP in other chelonian species, all of which have found higher WBC counts using the same or similar phloxine-based stains compared to EST [33–36]. The magnitude of disagreement between all methods increased as mean WBC count increased on corresponding Bland Altman plots, though this proportional bias was only statistically supported by Passing-Bablok regression for EST vs. NH (slope = 1.44 [1.03–1.83]). When the proportional bias between EST and NH is adjusted for via Passing-Bablok regression, the constant bias between the methods decreases substantially (intercept = -0.160 [-3.02–3.12] WBC x 10^9^/L). Given this and the relatively narrow LOA (12.64 [10.50–14.78] WBC x 10^9^/L), EST and NH showed the greatest agreement of all method pairs. In contrast, LP and NH exhibited the poorest agreement with the widest LOA (21.58 [18.00–25.16] WBC x 10^9^/L) and a lack of even basic positive correlation. Additionally, the difference in total WBC count by LP and both other methods was positively correlated with the proportion of lymphocytes and negatively correlated with the proportion of heterophils. This supports the supposition that LP overestimates total WBC count in high lymphocyte/low heterophil samples [24,35] and raises questions about its accuracy and utility in lymphocyte dominant species such as the radiated tortoise. Without an accepted gold standard or method-specific measures of precision, it is not possible to determine which method is the most accurate or precise. Nevertheless, our findings support the use of NH or EST over LP in radiated tortoises and emphasize the inadvisability of comparing results from different methods in individual patients or across populations.

The effect of time of sample collection on some hematological and biochemical analytes was an incidental finding in this study that warrants further investigation. Statistically significant differences in PM samples relative to AM samples included higher K, heterophil proportion, and heterophil:lymphocyte ratio and lower Ca and lymphocyte proportion. These differences may be due to circadian rhythm, temperature, or activity level, all of which have been show to affect certain hematology and biochemistry analytes in other reptile species [13,22,76–79]. For example, the changes in heterophil and lymphocyte proportions are consistent with shifts seen in some reptile species as glucocorticoids increase [80], which in turn has been shown to vary with temperature [81,82] and diurnally [83]. Similarly, K and Ca may be secondarily affected by the demonstrated effects of temperature and activity-level on blood gas and acid-base status [77,79,84]. As this study was not designed to evaluate the effects of time of day, reasons for observed differences are speculative. Future experiments that measure hematological and biochemical analytes in a time series with concurrent ambient and tortoise temperature, an index of activity level, and blood corticosterone would elucidate underlying etiologies and clinical implications of differences along these axes.

Certain limitations of this study should be considered when interpreting and applying our results. Reference intervals must be based on healthy individuals and while tortoises included in this study were apparently healthy based on physical examination and pathogen screening, subtler indicators of poor health such as changes in activity level and appetite may have been overlooked in this high volume rehabilitation setting. Additionally, biochemistry results were restricted by the dynamic range of the VetScan VS2 for certain analytes, imposing artificial limits on reference intervals and hampering the ability to detect effects of venipuncture site, sample artifacts, and time of day. By substituting out of range values with the value closest to the analyzer threshold rather than removing out of range values from analyses, these effects were reduced but not eliminated. Bile acids, UA, Alb, and Glob (which is calculated from Alb and TP) were most affected with results for these analytes below the reportable range for several or all tortoises. Other researchers have encountered similar constraints using the VetScan in alligator snapping turtles (*Macrochelys temminckii*) [85] and loggerhead turtles (*Caretta caretta*) [86,87]. As liver and renal disease are associated with elevated BA and UA, the VetScan and values reported here are likely useful for detecting clinical disease, but lack precision for ecophysiological research applications. In contrast, the current VetScan system cannot be recommended for measurement of Alb or Glob in this species for either clinical or research purposes with protein electrophoresis being the preferred method where resources allow [88,89]. For other biochemistry analytes, the VetScan remains a valuable tool for clinical care and research in chelonian populations where access to reference laboratories is limited, though species-specific validation is strongly recommended.

By determining population- and technique-specific hematology and biochemistry reference intervals and quantifying how these analytes vary between commonly used techniques, this study provides a valuable tool for improving care along the confiscation-to-release pathway critical to radiated tortoise conservation. These results also contribute foundational knowledge for future clinical research for this critically endangered species. We focused our primary efforts on the target population for release, subadults at the TCC, at a single time point. Further research is needed to characterize variation in hematology and biochemistry reference intervals across age groups and seasons within the rehabilitation population and to evaluate associations between these analytes, clinical outcomes, and post-release survival.

## Supporting information

S1 Table Subadult radiated tortoise biochemistry reference intervals and descriptive statistics in United States conventional units based on clinically healthy individuals living in a rehabilitation setting within their natural range. (DOCX)
