# Supplementary material for: Hematology and biochemistry of critically endangered radiated tortoises (Astrochelys radiata): Reference intervals in previously confiscated subadults and variability based on common techniques
Source: PLoS One. 2022 Mar 14;17(3):e0264111. doi: 10.1371/journal.pone.0264111 (PMC8920285; doi:10.1371/journal.pone.0264111)
Supplement: S1 Table — (DOCX) [file pone.0264111.s001.docx]

**Table 1: Subadult radiated tortoise biochemistry reference intervals and descriptive statistics in United States conventional units based on clinically healthy individuals living in a rehabilitation setting within their natural range.**

| ***Analyte*** | ***VS*** | ***n*** | ***Mean*** | ***SD*** | ***Median*** | ***Min*** | ***Max*** | ***p*** | ***Dist.*** | ***RI Method*** | ***RI*** | ***LRL***  ***90% CI*** | ***URL***  ***90% CI*** | ***Data excluded*** |
| --- | --- | --- | --- | --- | --- | --- | --- | --- | --- | --- | --- | --- | --- | --- |
| TS (g/dL) | All | 120 | 3.8 | 0.7 | 3.9 | 2.3 | 5.8 | 0.012 | NG | NP | 2.5–5.1 | 2.3–2.7 | 4.8–5.8 | N/A |
| TP (g/dL) | All | 120 | 2.9 | 0.5 | 2.9 | 1.9 | 4.3 | 0.210 | NG | NP | 1.9–4.2 | 1.9–2.1 | 3.7–4.3 | N/A |
| AST (U/L) | All | 120 | 81 | 43 | 68 | 31 | 342 | <0.001 | NG | NP | 39–218 | 31–44 | 166–342 | N/A |
| AST (U/L) | SC | 80 | 74 | 34 | 65 | 31 | 245 | <0.001 | NG | NP | 38–209 | 31–41 | 120–245 | N/A |
| AST (U/L) | Br | 40 | 95 | 55 | 81 | 47 | 342 | <0.001 | NG | NP | 47–339 | 47–51 | 194–342 | N/A |
| CK (U/L) | All | 53 | 660 | 626 | 487 | 193 | 4011 | <0.001 | NG | RT | 201–2455 | 184–229 | 1726–3618 | time to analysis > 5 hrs;  hem > 0 |
| CK (U/L) | SC | 39 | 478 | 235 | 452 | 193 | 1358 | 0.008 | NG | RT | 190–1072 | 0–211 | 898–1281 | time to analysis > 5 hrs;  hem > 0 |
| CK (U/L) | Br | 14 | 1165 | 1016 | 912 | 310 | 4011 | 0.008 | NG | N/A | N/A | N/A | N/A | time to analysis > 5 hrs;  hem > 0 |
| UA (mg/dL) | All | 120 | 0.4 | 0.1 | 0.3 | 0.3 | 0.8 | <0.001 | NG | NP | 0.29–0.70 | 0.29–0.29 | 0.70–0.80 | N/A |
| Glucose (mg/dL) | All | 90 | 57 | 12 | 57 | 35 | 104 | 0.477 | G | R | 33–81 | 28–37 | 76–85 | time to analysis > 5 hrs |
| Glucose (mg/dL) | SC | 57 | 53 | 10 | 53 | 35 | 73 | 0.417 | G | R | 33–72 | 31–37 | 68–76 | time to analysis > 5 hrs |
| Glucose (mg/dL) | Br | 33 | 65 | 12 | 64 | 36 | 104 | 0.183 | NG | RT | 42–93 | 37–49 | 83–103 | time to analysis > 5 hrs |
| Total Ca (mg/dL) | All | 120 | 11.9 | 1.3 | 11.8 | 8.6 | 16.1 | 0.049 | NG | NP | 9.4–14.9 | 8.6–9.6 | 13.8–16.1 | N/A |
| Phos (mg/dL) | All | 120 | 3.2 | 0.7 | 3.1 | 2.2 | 5.6 | <0.001 | NG | NP | 2.3–5.0 | 2.2–2.4 | 4.6–5.6 | N/A |
| Phos (mg/dL) | SC | 80 | 3.1 | 0.5 | 3.0 | 2.2 | 4.7 | 0.001 | NG | NP | 2.3–4.6 | 2.2–2.4 | 4.1–4.7 | N/A |
| Phos (mg/dL) | Br | 40 | 3.4 | 0.8 | 3.3 | 2.3 | 5.6 | <0.001 | NG | NP | 2.3–5.6 | 2.3–2.4 | 5.0–5.6 | N/A |
| Ca:Phos Ratio | All | 120 | 3.9 | 0.8 | 3.8 | 2.1 | 5.7 | 0.639 | G | R | 2.3–5.4 | 2.1–2.5 | 5.2–5.6 | N/A |
| Ca:Phos Ratio | SC | 80 | 4.0 | 0.8 | 3.9 | 2.2 | 5.7 | 0.609 | G | R | 2.5–5.5 | 2.3–2.7 | 5.3–5.7 | N/A |
| Ca:Phos Ratio | Br | 40 | 3.6 | 0.8 | 3.5 | 2.1 | 5.2 | 0.858 | G | R | 2.0–5.2 | 1.7–2.4 | 4.9–5.5 | N/A |
| K (mmol/L) | All | 77 | 6.4 | 0.9 | 6.5 | 4.3 | 8.4 | 0.553 | G | R | 4.5–8.2 | 4.2–4.8 | 7.9–8.5 | time to analysis > 8 hrs |
| Na (mmol/L) | All | 120 | 145.0 | 11.3 | 144.0 | 124.0 | 169.0 | 0.001 | NG | NP | 128–168 | 124–130 | 166–169 | N/A |

Results stratified by venipuncture site where statistically significant differences were found via generalized linear models. AST = aspartate transaminase, Br = brachial, Ca = calcium, CI = confidence interval, CK = creatine kinase, Dist. = distribution, G = Gaussian, hem = hemolysis (0, 1+, 2+, 3+), K = potassium, LRL = lower reference limit, Na = sodium, NG = Non-Gaussian, NP = Non-parametric, p = Anderson-Darling p-value, Phos = phosphorous, R = Robust, RT = Robust on Box Cox transformed data, SC = subcarapacial, SD = standard deviation, time to analysis = time from sample collection to biochemistry analysis, TP = total protein, TS = total solids, UA = uric acid, URL = upper reference limit, VS = venipuncture site. α = 0.3 for distribution tests per ASVCP Guidelines 2020.
